# From zebrafish heart jogging genes to mouse and human orthologs: using Gene Ontology to investigate mammalian heart development.

**DOI:** 10.12688/f1000research.2-242.v2

**Published:** 2014-02-10

**Authors:** Varsha K Khodiyar, Doug Howe, Philippa J Talmud, Ross Breckenridge, Ruth C Lovering

**Affiliations:** 1 Cardiovascular GO Annotation Initiative, Centre for Cardiovascular Genetics, Institute of Cardiovascular Science, University College London, London, WC1E 6JF, UK; 2 The Zebrafish Model Organism Database, University of Oregon, Eugene, OR, 97403-5291, USA; 3 Centre for Metabolism and Experimental Therapeutics, University College London, London, WC1E 6JF, UK

## Abstract

For the majority of organs in developing vertebrate embryos, left-right asymmetry is controlled by a ciliated region; the left-right organizer node in the mouse and human, and the Kuppfer’s vesicle in the zebrafish. In the zebrafish, laterality cues from the Kuppfer’s vesicle determine asymmetry in the developing heart, the direction of ‘heart jogging’ and the direction of ‘heart looping’.  ‘Heart jogging’ is the term given to the process by which the symmetrical zebrafish heart tube is displaced relative to the dorsal midline, with a leftward ‘jog’. Heart jogging is not considered to occur in mammals, although a leftward shift of the developing mouse caudal heart does occur prior to looping, which may be analogous to zebrafish heart jogging. Previous studies have characterized 30 genes involved in zebrafish heart jogging, the majority of which have well defined orthologs in mouse and human and many of these orthologs have been associated with early mammalian heart development.

We undertook manual curation of a specific set of genes associated with heart development and we describe the use of Gene Ontology term enrichment analyses to examine the cellular processes associated with heart jogging.  We found that the human, mouse and zebrafish ‘heart jogging orthologs’ are involved in similar organ developmental processes across the three species, such as heart, kidney and nervous system development, as well as more specific cellular processes such as cilium development and function. The results of these analyses are consistent with a role for cilia in the determination of left-right asymmetry of many internal organs, in addition to their known role in zebrafish heart jogging.

This study highlights the importance of model organisms in the study of human heart development, and emphasises both the conservation and divergence of developmental processes across vertebrates, as well as the limitations of this approach.

## Introduction

An understanding of heart development is important for the treatment of both congenital and acquired heart disease. The majority of heart development studies use model organisms for ethical and practical reasons. Transparent fish embryos, as well chick embryos, enable the developing heart to be studied in real time ^1^, and the mouse continues to be a key model organism used to investigate mammalian heart development ^2^. Although there is substantial evolutionary conservation in the development of left-right axis asymmetry, there is divergence between species ^3^. The earliest events in mammalian heart development are of great interest, but are poorly understood relative to externally developing organs, due to practical constraints.

For the majority of developing vertebrate embryos left-right asymmetry is controlled by a ciliated region; the left-right organizer node in the mouse and human, and the Kuppfer’s vesicle in the zebrafish ^4, 5^. In the zebrafish, laterality cues from the Kuppfer’s vesicle determine asymmetry in the developing heart, and consequently the direction of heart jogging and heart looping. At 24 hours post-fertilization (hpf) the symmetrical zebrafish heart tube is displaced relative to the dorsal midline, with a leftward ‘jog’. At 36hpf the heart tube then loops to the right to create the asymmetric heart ^5, 6^. Cilia within the Kuppfer’s vesicle are known to be instrumental in establishing left-right asymmetry and consequently play a significant role in determining the direction of heart jogging ^7^ and heart looping ^8^. However, a failure of heart jogging does not necessarily imply that there will be a failure in heart looping, and vice versa. In addition, asymmetric cell migration has been implicated as a key factor in the process of heart jogging ^9– 13^. Several of the genes involved in zebrafish heart jogging have been identified from mutation, morpholino and functional complementation studies ^6, 10, 13– 26^.

We sought to determine whether the use of Gene Ontology (GO) annotation could offer mechanistic clues to early mammalian heart development. GO is a controlled vocabulary that is used to describe gene product function ^27^. GO describes three aspects of a gene product’s biology: the *biological process* that the gene product is involved in, the specific *molecular function* of the gene product and the *cellular component* that the gene product is located in. GO terms are associated in a directed acyclic graph (DAG), and thus have defined relationships to each other.

The process of heart looping has been described in a variety of higher eukaryotes ^2, 28, 29^, and the occurrence of dextral-looping, the early phase of heart looping, appears to be conserved from zebrafish to chicken to humans. In addition, many congenital heart abnormalities, such as dextrocardia and isomerisms are thought to be due to abnormal heart looping ^2, 30^ and ciliary dysfunction has been associated with 50% of patients with congenital heart disease and heterotaxy ^31^. However, the process of heart jogging has only been described in zebrafish ^6^. Biben and Harvey describe a leftward shift of the developing mouse caudal heart prior to looping, which may be analogous to heart jogging in zebrafish ^28^, but to our knowledge this has not been investigated further, and heart jogging is not considered to occur in mammals. Consequently, when the ontology describing heart development was expanded ^32^, limitations were included to prevent the association of the GO term ‘heart jogging’ to mammalian gene products ^33^. However, an absence of evidence is not evidence of absence, hence it remains a possibility that heart jogging also occurs in mammalian systems.

Although there has been substantial progress in heart development research ^1, 3, 4, 29^, there are clearly gaps in our understanding of early heart development, particularly in the mammal. Functional enrichment analysis of genes known to be involved in zebrafish heart jogging, and also of the human and mouse orthologs of these zebrafish heart jogging genes, identifies many conserved biological processes, functions and cellular locations across these three species. The results of these analyses support the role of cilia in symmetry breaking and the importance of cell signalling in early heart development.

## Methods

### Generation of the list of zebrafish heart jogging genes

A list of 30 zebrafish genes that affect heart jogging was compiled using a variety of approaches. Twelve zebrafish proteins were identified as they were already annotated to the ‘heart jogging’ GO terms, the remaining 18 proteins were then identified using the ZFIN ( http://zfin.org/) Site Search, with the search phrase 'heart jogging', and filtering using the 'Expression/Phenotypes' category. This search retrieves figures from papers that have ‘heart jogging’ in the figure legend, and thus are likely to be describing specific zebrafish genes (and proteins) involved in this process. Many of these genes had not yet been curated with GO terms. Each of the papers identified in this way were reviewed; of the 23 zebrafish genes identified in these papers five (Bmpr1aa, Tbx1, unm_hu119, unm_hu202, unm_hu304) were eliminated, as none of these papers provided experimental evidence for the involvement of these genes in heart jogging. This left 30 zebrafish proteins with strong evidence for a role in the heart jogging process ( Table 1). The experimental evidence describing the association of each gene to the process of heart jogging was manually reviewed, to ensure consistent criteria were applied.

**Table 1.** Proteins included in zebrafish ‘jogging’ gene list and the human and mouse ‘jogging ortholog’ gene lists. The evidence for these 30 zebrafish proteins having a role in heart jogging comes from mutant, morpholino or functional complementation studies, as described in the associated publications.

*Zebrafish gene symbol* *(protein ID)*	*Human gene symbol* *(protein ID)*	*Mouse gene symbol* *(protein ID)*
*acvr1l* ^6^ (Q9DGI6)	*ACVRL1* (P37023)	*Acvrl1* (Q61288)
*apc* ^57^ (F1QN37)	*APC* (P25054)	*Apc* (Q61315)
*bmp4* ^6, 13^ (O57574)	*BMP4* (P12644)	*Bmp4* (P21275)
*bmp7a* ^6^ (Q9PTF9)	*BMP7* (P18075)	*Bmp7* (P23359)
*vbmpr2a* ^20^ (Q288P3)	*BMPR2* (Q13873)	*Bmpr2* (O35607)
*bmpr2b* ^20^ (Q288P2)		
*camk2a* ^14^ (Q32PV2)	*CAMK2A* (Q9UQM7)	*Camk2a* (P11798)
*camk2b2* ^14^ (E7F012)	*CAMK2B* (Q13554)	*Camk2b* (P28652)
*camk2g1* ^14^ (Q4V9P8)	*CAMK2G* (Q13555)	*Camk2g* (Q923T9)
*ccdc103* ^6^ (Q6DGB6)	*CCDC103* (Q8IW40)	*Ccdc103* (Q9D9P2)
*ccdc40* ^6^ (Q56A40)	*CCDC40* (Q4G0X9)	*Ccdc40* (Q8BI79)
*cobl* ^23^ (I1X3U9)	*COBL* (O75128)	*Cobl* (Q5NBX1)
*dand5* ^15^ (Q76C29)	*DAND5* (Q8N907)	*Dand5* (Q76LW6)
*dnaaf1* ^6, 10^ (Q7ZV84)	*DNAAF1* (Q8NEP3)	*Dnaaf1* (Q9D2H9)
*dub* ^22^ (Q0P484)	*RCSD1* (Q6JBY9)	*Rcsd1* (Q3UZA1)
*fgfr2* ^18^ (Q8JG38)	*FGFR2* (P21802)	*Fgfr2* (P21803)
*foxh1* ^6, 58^ (Q9I9E1)	*FOXH1* (O75593)	*Foxh1* (O88621)
*foxj1a* ^25^ (Q08CI2)	*FOXJ1* (Q92949)	*Foxj1* (Q61660)
*foxj1b* ^25^ (F1R8Z9)		
*fzd2* ^22^ (Q90YL7)	*FZD2* (Q14332)	*Fzd2* (Q9JIP6)
*gsk3b* ^17^ (Q9IBD2)	*GSK3B* (P49841)	*Gsk3b* (Q9WV60)
*has2* ^13^ (Q9DG41)	*HAS2* (Q92819)	*Has2* (P70312)
*lrrc6* ^6^ (B3DH20)	*LRRC6* (Q86X45)	*Lrrc6* (O88978)
*nipbla* ^21^ (F5HSE3)	*NIPBL* (Q6KC79)	*Nipbl* (Q6KCD5)
*Niplblb* ^21^ (F1QBY1)		
*nkd1* ^24^ (Q2TJA6)	*NKD1* (Q969G9)	*Nkd1* (Q99MH6)
*nphp3* ^26^ (P0CI65)	*NPHP3* (Q7Z494)	*Nphp3* (Q7TNH6)
*pkd2* ^6^ (Q6IVV8)	*PKD2* (Q13563)	*Pkd2* (O35245)
*ptpn11a* ^16^ (Q7ZW17)	*PTPN11* (Q06124)	*Ptpn11* (P35235)
*southpaw* ^10, 19^ (Q7ZZT5)	no mammalian orthologs	

### Generation of the list of human and mouse ‘jogging ortholog’ genes

The HUGO Gene Nomenclature Committee Comparison of Orthology Predictions (HCOP) search tool ( http://www.genenames.org/cgi-bin/hcop.pl) was used to identify the closest possible human and mouse ortholog for each of the 30 zebrafish genes. HCOP displays predictions from 11 homology prediction tools, including EnsemblCompara, Homologene and Inparanoid ^34^. For all but one gene, *southpaw*, HCOP returned human or mouse homologs for the zebrafish genes. The lack of a close mammalian ortholog of *southpaw* was confirmed with a UCSC BLAT analysis against the human and mouse genomes ^35^. BLAST analysis ^36^ showed that the closest possible human and mouse homolog for the zebrafish *southpaw* gene was *Nodal* (33% identity). Indeed, both *southpaw* and *nodal* are specifically expressed in the left lateral plate mesoderm ^5, 37^ and knockdown of murine *Nodal* in this region leads to a disruption of cardiac asymmetry, as does injection of *southpaw* morpholinos, suggesting a functional orthology between *southpaw* and *Nodal* ^5, 37^. However a reciprocal HCOP search showed that the zebrafish genes *nodal-related 1* and *2* are the closest orthologs of human *NODAL*. Hence we have not included a human or mouse ortholog for zebrafish *southpaw* ( Table 1). Three pairs of zebrafish paralogs ( *bmpr2a*/ *bmpr2b*; *foxj1a*/ *foxj1b*; *nipbla*/ *nipblb*) have a single corresponding ortholog in human and mouse. Therefore, there are 26 human and 26 mouse orthologs to the 30 zebrafish genes identified as relevant to zebrafish heart jogging ( Table 1).

### Gene ontology annotation

The human ‘jogging ortholog’ genes were fully manually annotated, by an experienced GO curator ^38^. Individual PubMed queries were run for each gene using the approved human gene symbol and filtering on ‘human’. To achieve full annotation, all of the relevant publications (a total of 232) containing unique functional data for each gene were annotated, regardless of the specific biology described in each paper. This approach enabled consistent annotation of all experimental data relating to each gene, thus ensuring an unbiased overview of any common processes associated with these genes. In addition, the GO term ‘heart looping’ was associated with a ‘jogging ortholog’ human gene if dextrocardia or *situs inversus totalis* phenotypes had been associated with a mutation in the gene, in order to follow the generally agreed view that leftward heart looping will have resulted in these phenotypes ^2^.

### Functional enrichment analysis

The Mouse Genome Informatics functional enrichment tool VLAD (VisuaL Annotation Display; http://proto.informatics.jax.org/prototypes/vlad-1.0.3/) was used to look for overrepresentation of GO terms in each gene list relative to the whole genome of the organism. The annotation datasets used for the analysis were zfin (4th March 2013), goa_human (5th March 2013) and mgi (7th March 2013) for the zebrafish, human and mouse analyses respectively, and the ontology dataset used was dated 10th March 2013. The query gene lists (as UniProt IDs) were pasted into the ‘Query Set’ field, the ‘Universe Set’ field was left blank (to specify all genes in species specific annotation file) and the ‘Display Settings’ options selected were ‘pruning threshold’:3 and ‘collapsing threshold’:6. No evidence codes were excluded from the analyses. For this analysis the total number of genes (universe set size) having annotations in the *biological process* ontology were 14,577, 30,441 and 24,813 for zebrafish, human and mouse respectively. In line with common practice, when using functional analysis tools, enriched GO terms with 1 or 2 associated query genes were excluded from the final results table.

### Creation of an ‘early heart development’ mouse gene list

A list of 103 mouse genes likely to play a role in early heart development was created by combining gene lists derived from three sources: The Mouse Genome Informatics Mammalian Phenotype Ontology browser http://www.informatics.jax.org/searches/MP_form.shtml ^39^, the QuickGO browser http://www.ebi.ac.uk/QuickGO/ ^40^ and the ‘jogging ortholog’ gene list described above (see Mousegenelist.csv in Data File). The Mammalian Phenotype Ontology browser was queried for genotypes annotated with the terms ‘abnormal direction of heart looping’, ‘ *situs inversus totalis*’, ‘dextrocardia’ and ‘mesocardia’, creating a list of 180 genotypes with an associated gene. Due to the multiple phenotypes associated with each of these genotypes only 58 genes were identified through this approach, and of these only 5 overlap with the 26 ‘jogging ortholog’ genes. Thirty-five genes were identified by filtering on the GO term ‘determination of heart left/right asymmetry’ and its child terms, the evidence code IMP (Inferred by Mutant Phenotype), and the mouse taxon. Of these only two are also present in the ‘jogging ortholog’ gene lists and 11 are present in the phenotype gene list. Twenty-six mouse ‘jogging ortholog’ genes were added to this combined gene list, and any duplicated genes were removed.

## Results

### Annotation of the zebrafish heart jogging genes and the human ‘jogging ortholog’ genes

Thirty zebrafish genes were annotated to the GO term ‘heart jogging’ or one of its child terms based on experimental data from the literature ( Table 1). Human and mouse orthologs of these genes were identified, as described in the Methods section, resulting in a list of 26 mammalian ‘jogging orthologs’.

The human ‘jogging ortholog’ genes were then fully annotated with GO terms based on published experimental data. All manual annotations to the human, mouse and zebrafish genes can be visualized with the QuickGO Gene Ontology browser http://tinyurl.com/humanortholog, http://tinyurl.com/mouseortholog and http://tinyurl.com/zebrafishgenes.

### Functional enrichment analysis

The zebrafish heart jogging gene list and the human and mouse ‘jogging ortholog’ gene lists were analysed using the VLAD enrichment tool. This identified 155 *biological process* GO terms that were significantly enriched in the zebrafish (see Human_data.csv in Data File), 431 in the human (see Human_data.csv in Data File) and 402 in the mouse (see Mouse_data.csv in Data File) gene lists. The enriched GO terms from all three species were grouped into five biological areas: Development, Patterning, Cellular Process, Signalling and Movement. The relative enrichment of key GO terms from each area was compared across all three species (see Biological_process_summary.csv in Data File; summarized in Table 2).

**Table 2.** Comparison of enriched Gene Ontology terms across orthologous gene lists from zebrafish, human and mouse. The enriched GO terms were grouped into specific ontology areas, with a selection of more specific child term (preceded with a dash) also included. The full list of grouped GO terms can be found in Table S4, which also shows the genes annotated to each term from each of the three species. k: the number of genes in each gene list annotated to the GO term; M: the number of genes in the species proteome annotated to the GO term.

*Gene Ontology terms*	*Zebrafish*			*Human*			*Mouse*		
	*k*	*M*	*k/M as %*	*k*	*M*	*k/M as %*	*k*	*M*	*k/M as %*
**DEVELOPMENT**									
GO:0032502 developmental process	30	2357	1.3%	24	6803	0.4%	22	3945	0.6%
- GO:0009888 tissue development	30	673	4.5%	17	1849	0.9%	14	1138	1.2%
- GO:0072358 cardiovascular system development	30	487	6.2%	15	1095	1.4%	10	679	1.5%
- GO:0001944 vasculature development	-	-	-	7	666	1.1%	7	436	1.6%
- GO:0007507 heart development	30	268	11.2%	13	634	2.1%	8	390	2.1%
- GO:0001947 heart looping	22	83	26.5%	7	68	10.3%	-	-	-
- GO:0007399 nervous system development	8	700	1.1%	15	2802	0.5%	12	1486	0.8%
- GO:0072001 renal system development	6	100	6.0%	9	386	2.3%	6	200	3.0%
- GO:0007423 sensory organ development	-	-	-	10	738	1.4%	8	480	1.7%
- GO:0048736 appendage development	4	123	3.3%	4	237	1.7%	4	159	2.5%
- GO:0050793 regulation of developmental process	6	267	2.2%	14	2383	0.6%	14	1542	0.9%
**PATTERNING**									
GO:0007389 pattern specification process	30	435	6.9%	15	644	2.3%	12	414	2.9%
- GO:0009799 specification of symmetry	30	177	16.9%	10	147	6.8%	7	90	7.8%
- GO:0061371 determination of heart left/right asymmetry	30	97	30.9%	8	71	11.3%	4	48	8.3%
**CELLULAR PROCESS**									
GO:0071840 cellular component organization or biogenesis	12	1124	1.1%	17	5991	0.3%	15	3306	0.5%
- GO:0030030 cell projection organization	10	274	3.6%	12	1311	0.9%	11	644	1.7%
- GO:0051128 regulation of cellular component organization	-	-	-	10	1894	0.5%	10	1293	0.8%
- GO:0031344 regulation of cell projection organization	-	-	-	5	452	1.1%	5	311	1.6%
GO:0006468 protein phosphorylation	8	696	1.1%	8	842	1.0%	9	748	1.2%
- GO:0001932 regulation of protein phosphorylation	-	-	-	8	1124	0.7%	8	722	1.1%
GO:0006357 regulation of transcription from RNA polymerase II promoter	8	166	4.8%	10	1939	0.5%	12	1268	0.9%
GO:0042127 regulation of cell proliferation	5	70	7.1%	9	1769	0.5%	9	1123	0.8%
GO:0007049 cell cycle	-	-	-	9	1644	0.5%	8	915	0.9%
- GO:0051726 regulation of cell cycle	-	-	-	6	934	0.6%	6	538	1.1%
**SIGNALLING**									
GO:0023052 signaling	12	2131	0.6%	16	6682	0.2%	16	4568	0.4%
- GO:0023051 regulation of signaling	7	521	1.3%	16	3227	0.5%	16	1931	0.8%
GO:0050896 response to stimulus	-	-	-	20	10767	0.2%	21	6679	0.3%
- GO:0048583 regulation of response to stimulus	7	557	1.3%	15	3799	0.4%	15	2140	0.7%
**MOVEMENT**									
GO:0007017 microtubule-based process	6	188	3.2%	6	873	0.7%	4	358	1.1%
GO:0040011 locomotion	10	289	3.5%	10	1448	0.7%	8	753	1.1%
- GO:0040012 regulation of locomotion	-	-	-	6	657	0.9%	5	473	1.1%
- GO:2000145 regulation of cell motility	-	-	-	6	608	1.0%	5	439	1.1%
- GO:0016477 cell migration	8	200	4.0%	6	931	3.6%	5	504	1.0%

***Enrichment of heart development terms.*** As expected there was a significant enrichment of developmental process terms in all three gene lists, including an enrichment of the GO term ‘heart development’. However, there was also enrichment of terms such as ‘renal system development’ and ‘nervous system development’, indicating the role of these proteins in regulating the development of a range of organ systems and tissues. These data analyses also show an enrichment of terms describing specific, but universal, cellular processes, such as signalling and regulation of transcription ( Table 2). These terms represent essential aspects of development, but are grouped discretely due to their roles in many other biological processes.

‘Pattern specification’, described in GO as a ‘developmental process that results in the creation of defined areas or spaces within an organism to which cells respond and eventually are instructed to differentiate’ and several of its more specific child terms (such as ‘specification of symmetry’), were also enriched in all three gene lists. Within the symmetry ontology, the GO term ‘determination of heart left/right asymmetry’ is annotated to all 30 genes in the zebrafish jogging gene list, however, it is only associated with 8 and 4 jogging ortholog genes in the human and mouse respectively. Of the 97 zebrafish genes associated with ‘determination of heart left/right asymmetry’ 31% are also present in the zebrafish jogging gene list. In contrast, only 11% of the human and 8% of the mouse genes associated with this term are also ‘jogging orthologs’. These results confirm an overlap in the functional role of the zebrafish jogging genes and the human and mouse orthologs in the determination of heart left/right symmetry. However, this relatively low level of overlap may reflect the limitations of model organism and human research in this area, rather than a lack of functional conservation of these genes.

In addition, there were some differences in the developmental terms that were enriched between species. For example, the GO terms ‘vasculature development’ and ‘sensory organ development’ are enriched in both the human and mouse ‘jogging ortholog’ gene lists ( Table 2), but neither of these processes are enriched in the zebrafish jogging ortholog genes. This difference may reflect the type of experiments zebrafish are used for, rather than reflecting a difference between zebrafish and mammals in the genes required for these developmental processes.

**Figure 1.**
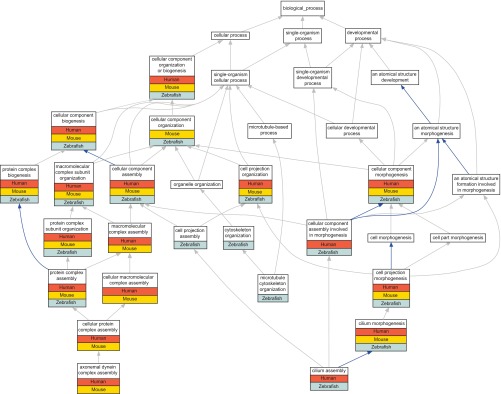
View of relationships between enriched terms from the cellular component organization or biogenesis ontology. The GONUTs view of relationships between enriched terms from the cellular component organization or biogenesis ontology ^84^. The grey arrows are used where a term has an ‘is a’ relationship to its parent term, the blue arrows indicate a ‘part of’ relationship. The bars below each GO term indicates which of these terms are enriched in the zebrafish ‘jogging’ gene list, and the human and mouse ‘jogging ortholog’ gene lists.

***Enrichment of cilia terms.*** Terms in the cellular component organization or biogenesis ontology were enriched across all three gene lists ( Table 2 and Biological_process_summary.csv in Data File). Specifically there was an enrichment of terms describing ‘cilium morphogenesis’ and ‘protein complex assembly’ ( Figure 1). Within each of these, some more specific terms were enriched, for example the human and mouse ‘jogging ortholog’ gene lists were enriched for the term ‘axonemal dynein complex assembly’, whilst the zebrafish and human gene lists showed an enrichment of the term ‘cilium assembly’.

Terms such as ‘regulation of cell projection organization’ were also enriched in the human and mouse ‘jogging ortholog’ gene lists. ‘Regulation’ terms have a ‘regulates’ relationship with the relevant processes; for example the term ‘positive regulation of cell projection organization’ has a ‘positively_regulates’ relationship to the term ‘cell projection organization’. In GO an important benefit of building a DAG, rather than a flat-list of controlled vocabulary terms, is that relationships can be used to make inferences from one term to another. However, the VLAD enrichment tool does not automatically create a transitive relationship between ‘regulation’ terms and the processes or functions they regulate. Consequently genes annotated to a ‘regulation’ term will not be associated with the regulated process term (unless there is an independent annotation to the process term). It is also important to recognise that it can be difficult for a curator to choose between annotating to the *biological process* itself, or to the term describing the regulation of that *biological process*, based on the published experimental data. Therefore, to get a full picture of the genes involved in a process, including the genes that regulate the process, it is necessary to combine the genes annotated to GO terms describing both the ‘process’ and the ‘regulation of the process’. For example, 10 zebrafish, 12 human and 14 mouse genes within the ‘jogging ortholog’ gene lists are annotated to either ‘cell projection organization’ or ‘regulation of cell projection organization’ (or children of these terms). This represents 33%, 40% and 47% of these zebrafish, human and mouse ‘jogging’ gene lists respectively, indicating that the process of cell projection organization is an important function for this group of genes. In addition, many of the ‘jogging’ and ‘jogging ortholog’ genes annotated to ‘cell projection organization’ terms have also been annotated to the cellular component term ‘cell projection’ (7, 11, 13, genes in zebrafish, human and mouse, respectively, see Data File). The enrichment of the *biological process* term ‘cell projection organization’ and *cellular component* term ‘cell projection’ within these gene lists is consistent with the key role of cilia located in the node/Kuppfer’s vesicle to determine heart left/right asymmetry in all three species.

***Enrichment of cell migration terms.*** Cell migration also plays a key role in the establishment of the heart cone, heart jogging and heart looping ^9, 10^ and enrichment of the GO term ‘cell migration’ is seen in the ‘jogging’ gene lists of all three species ( Table 2 and Biological_process_summary.csv in Data File). Lenhart *et al.* (2013) ^9^ identified *FoxH1*, *spaw*, *Bmp4*, *Lefty2* and *Has2* as essential to the asymmetric cell migration that leads to heart jogging. However, our literature review suggests that some genes may have functions in both cilia assembly, within the Kuppfer’s vesicle, and cell migration. For example, thymocytes from *Foxj1* transgenic mice display defective migration ^41^, whereas *Foxj1*-null mice are defective in ciliogenesis ^42^. Similarly, in zebrafish, *Fzd2* has been shown to play a role in cilium assembly ^22^ as well as pancreatic insulin-cell migration ^43^. Consequently, further investigations into the role of these genes in heart jogging cell migration may provide further insight into this process.

### Co-annotation of heart development associated genes

In order to investigate the contribution of individual genes in the multiple processes associated with early heart development we created human and mouse heart development gene lists and examined the associated GO *biological processes* terms. A list of 103 mouse genes with roles in early heart developmental processes was created by merging the three gene lists created using the Mouse Genome Informatics phenotype browser, the QuickGO browser as well as the ‘jogging ortholog’ gene list (Mousegenelist.csv in Data File).

GO captures a range of biological processes that a single gene is involved in. By comparing the overlap between the GO terms associated with specific gene lists it is possible to see what cellular mechanisms are likely to be contributing to the various heart developmental processes. Using the QuickGO browser, genes in the zebrafish ‘heart jogging’ gene list, which were associated with the GO terms ‘heart looping’, ‘signal transduction’, ‘cell migration’ and ‘cell projection organization’ (and all child terms, including ‘regulation’ terms), were downloaded, as well as the genes associated with these terms that were also present in the mouse ‘early heart development’ gene list (Mousegenelist.csv in Data File).

**Figure 2.**
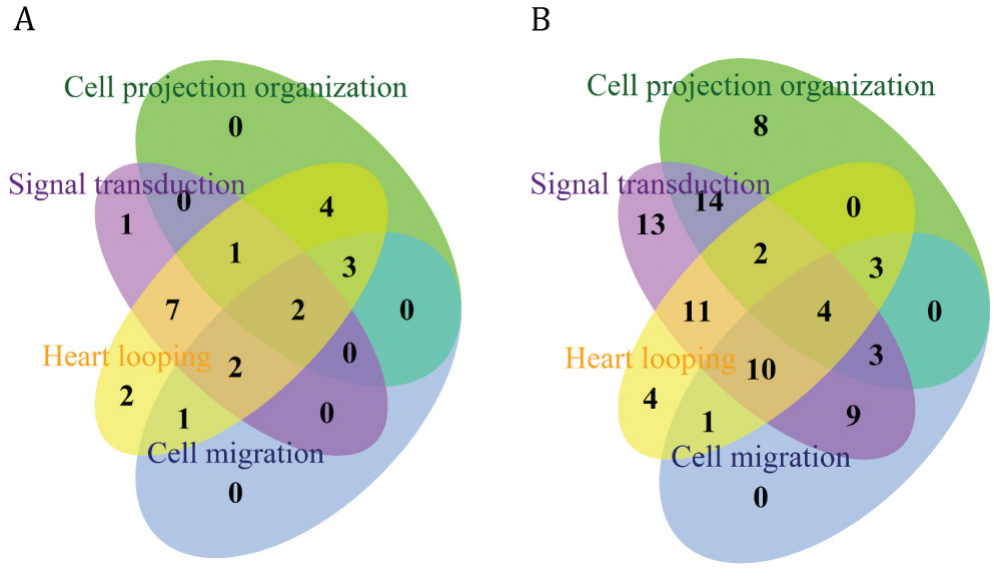
Venn diagrams describing the multiple roles of genes associated with heart development. Venn diagrams showing the overlap between the GO terms associated with **A**) the zebrafish ‘heart jogging’ gene list (30 genes) and **B**) the mouse combined heart development gene list (103 genes).

In the zebrafish ‘heart jogging’ gene list a similar proportion of the genes have the potential to play a role in cell projection organisation (10 genes), cell migration (8 genes) and signal transduction (13 genes) ( Figure 2A). In the list of 103 mouse genes that are associated with early heart development, either by phenotype, annotation or homology to the zebrafish ‘heart jogging’ gene list, 82 have been annotated to the GO term heart looping. In contrast to the zebrafish ‘jogging’ gene list, signal transduction appears to play a major role in the mouse early heart development, with 27 genes associated with both signal transduction and heart looping, whereas only 18 and 9 genes, respectively, are associated with cell migration and cell projection organization ( Figure 2B). These results fit well with what is known about these gene lists. The zebrafish ‘jogging’ gene list defines a group of genes whose functions are required very early in heart development, when the role of cilia in symmetry breaking initiates the heart jogging process. Whereas, in the mouse ‘early heart development’ gene list the genes included have roles in heart looping, which is developmentally later event than heart jogging. Therefore, although the initial events associated with breaking of left-right symmetry are represented within this gene list, the genes involved in the later process of ensuring the complex looping of the heart tube, through controlled signalling and cell migration, contribute to a large proportion of this list.

### Human disease phenotypes associated with the ‘jogging ortholog’ genes

While annotating the 26 human ‘jogging ortholog’ genes we noticed that almost half of these genes have not been associated with a specific disease phenotype ( Table 3). However, of the 26 genes examined, mutations in 14 had been associated with a disease phenotype, a fifth of which were ciliopathies. Dextrocardia or *situs inversus totalis* (reversal or mirroring of the major visceral organs) was associated with 6 of the human ‘jogging ortholog’ genes. Location of the heart on the right side (rather than the left) is generally agreed to be the result of left-handed, instead of right-handed looping of the heart tube in early embryogenesis ^2^. The association of these ‘jogging ortholog’ genes with heart looping defects confirm that there is conserved functional homology between at least some of these orthologous zebrafish and human genes in the very early stages of heart development, which lead to the initial heart asymmetry. All four of ciliopathy-associated ‘jogging orthologs’ were also described as associated with *situs inversus totalis*, confirming the conserved role of these genes in the cilia within the symmetry determining left-right organizer.

**Table 3.** Diseases associated with the human ‘jogging ortholog’ genes. The associated diseases are described in the listed publications.

*Human gene symbol* *(protein ID)*	*Heart relevant phenotype*	*Other associated phenotypes*
***ACVRL1* (P37023)**	-	Hereditary haemorrhagic telangiectasia type 2 (HHT2) ^59^, HHT2 with pulmonary hypertension ^60^
***APC* (P25054)**	-	Familial adenomatous polyposis coli-1 ^61^
***BMP4* (P12644)**	-	Microphthalmia, syndromic 6 ^62^, orofacial cleft 11 ^63^
***BMP7* (P18075)**	-	-
***BMPR2* (Q13873)**	-	Pulmonary hypertension ^64^
***CAMK2A* (Q9UQM7)**	-	-
***CAMK2B* (Q13554)**	-	-
***CAMK2G* (Q13555)**	-	-
***CCDC103* (Q8IW40)**	Dextrocardia, *situs inversus totalis* ^65^	Ciliary dyskinesia, primary, 17 ^65^
***CCDC40* (Q4G0X9)**	*Situs inversus totalis* ^66^	Ciliary dyskinesia, primary, 15 ^67^, Kartagener’s Syndrome ^66^
***COBL* (O75128)**	-	-
***DAND5* (Q8N907)**	-	-
***DNAAF1* (Q8NEP3)**	*Situs inversus totalis* ^68, 69^	Ciliary dyskinesia, primary, 13 ^68, 69^
***FGFR2* (P21802)**	-	Several craniosynostosis ^70, 71^, see OMIM for more information
***FOXH1* (O75593)**	Ventricular septal defect ^72^, transposition of the great arteries ^54^	-
***FOXJ1* (Q92949)**	-	-
***FZD2* (Q14332)**	-	-
***GSK3B* (P49841)**	-	-
***HAS2* (Q92819)**	-	-
***LRRC6* (Q86X45)**	*Situs inversus totalis* ^73^	Ciliary dyskinesia, primary, 19, Kartagener’s Syndrome ^73^
***NIPBL* (Q6KC79)**	Cardiac septal defects (not confirmed as associated with NIPBL mutations) ^74^	Cornelia de Lange syndrome 1 ^74, 75^
***NKD1* (Q969G9)**	-	Colorectal adenocarcinoma ^76^
***NPHP3* (Q7Z494)**	*Situs inversus totalis* ^77^	nephronophthisis type 3 ^78^, Meckel syndrome type 7 ^77^, renal-hepatic-pancreatic dysplasia ^77, 79^
***PKD2* (Q13563)**	Dextrocardia, *situs inversus totalis* ^53^	Polycystic kidney disease 2 ^53, 80^
***PTPN11* (Q06124)**	atrioventricular canal defects ^81^	juvenile myelomonocytic leukemia ^82^, LEOPARD syndrome ^81^, Noonan syndrome ^81, 83^
***RCSD1* (Q6JBY9)**	-	-

Significantly enriched GO terms in heart jogging genes in zebrafish and their human and mouse orthologs Zebrafish data: GO terms significantly enriched in zebrafish ‘heart jogging’ gene list. The GO biological process, molecular function and cellular component terms that are highly enriched in the zebrafish ‘jogging’ gene list. The GO terms are listed in order of decreasing p-values and the ‘universe set’ size included in the functional analysis using VLAD is 14,577.
Human data: GO terms significantly enriched in human orthologs of zebrafish ‘heart jogging’ genes. The GO biological process, molecular function and cellular component terms that are highly enriched in the human ‘jogging ortholog’ gene list. The GO terms are listed in order of decreasing p-values and the ‘universe set’ size included in the functional analysis using VLAD is 30,441.
Mouse data: GO terms significantly enriched in mouse orthologs of zebrafish ‘heart jogging’ genes. The GO biological process, molecular function and cellular component terms that are highly enriched in the mouse ‘jogging ortholog’ gene list. The GO terms are listed in order of decreasing p-values and the ‘universe set’ size included in the functional analysis using VLAD is 24,813.
Biological process summary: Summary of all enriched biological process GO terms across all three species. The enriched biological process GO terms are grouped into general biological areas (‘development’, ‘patterning’, ‘cellular process’, ‘signaling’, ‘movement’ and ‘high level terms’, text in upper case). Each area is subdivided into more specific ontologies, based on the Gene Ontology structure. A subset of the biological process GO terms and data are reported in Table 2 in the main article. High-level terms were not included in any further discussion, as these terms are not specific enough to be informative. k: the number of genes in each gene list annotated to the GO term; M: the number of genes in the species proteome annotated to the GO term. Starred genes are annotated directly to the enriched term. Non-starred genes are annotated to a ‘child’ of the enriched term. DAR: zebrafish, HUM: human, MUS: mouse.
Mouse gene list:‘Early heart development’ mouse gene list. The evidence that these 103 mouse genes have a role in the early stages of heart development comes from their orthology to the zebrafish ‘heart jogging’ gene list, from phenotypes associated with specific genotypes, or annotation to the GO term ‘determination of heart left/right asymmetry’ or it’s child terms (see method section in main article for full details).

## Discussion

We have used GO to annotate the key genes involved in zebrafish heart jogging and their human and mouse orthologs. Heart jogging is not a process that is thought to occur in mammals. However, these genes are conserved between species and play essential roles in many developmental processes. The information available about these genes in several diverse species can be used to shed light on the roles of these genes and possible mechanisms in heart jogging and other heart developmental processes. Our analyses are in agreement with the well described essential role of cilia in early development ^4, 5, 31^, with a third of the zebrafish ‘heart jogging’ genes associated with the *biological process* ‘cell projection organization’ ( Table 2).

However, it is also important to recognise that although there is considerable evidence for conserved mechanisms of heart development across vertebrates there are also areas of divergence ^44^. For example, in the mouse, zebrafish and *Xenopus* the rotation of cilia is responsible for the early asymmetric gene expression pattern around the left-right organizer, whereas cilia do not play a role in symmetry breaking in the chicken or pig ^44^.

The early phases of heart development are particularly difficult to study in mammals, however various approaches are enabling progress in this area ^2, 29, 45, 46^ and using phenotype, annotation and orthology data we have created a list of 103 genes with a putative role in early mouse heart developmental processes. Furthermore, the phenotypes associated with experimentally generated mutant mice provide further clues to the likely role of these genes in human heart development; the genes associated with *situs inversus totalis* phenotypes are most likely to have functional roles within the node. Conversely, genes not associated with *situs inversus totalis* but associated with an abnormal direction of heart looping, dextrocardia or mesocardia are likely to be involved in the response of the embryonic heart tube to the left/right asymmetry signals. This is not a completely reliable interpretation, for example mutations in the transcription factor *Pitx2* lead to mice with *situs inversus totalis*, however, *Pitx2* is expressed in the left lateral plate and its continued asymmetric expression is necessary for asymmetric morphogenesis of most visceral organs ^44^. The mouse knockout consortia data ^47^ will continue to help with the identification of additional early heart development genes, and informed interpretation of these phenotypes will make it possible to separate those genes likely to be associated with the node from those with functions within the heart tube.

In humans, defects in early heart development are likely to result in spontaneous abortion and therefore many genes required for early heart development will go undetected ^48^. Consequently, human embryos with heart defects, which develop to full term, represent the less severe end of the spectrum. Mutations in several human genes have now been identified as causative of abnormal heart looping, such as *ACVR2B*, *LEFTY2*, *GJA1* and *ZIC3* ^49– 52^, and some of the ‘jogging ortholog’ genes ( *CCDC103*, *CCDC40*, *DNAAF1*, *LRRC6*, *NPHP3* and *PKD2*) are also associated with heart looping defects. Thus providing evidence to support an involvement of these genes in left-right asymmetry determination in the heart. Furthermore, mutations in some of the ‘jogging ortholog’ human genes, *FOXH1* and *PTPN11*, are associated with heart septal defects in humans, which seems to imply that in individuals with these mutations early heart developmental processes have proceeded normally, suggesting that, contrary to their role in zebrafish, these genes may not be involved in the early stages of human heart development. However, there are other possible reasons why there is a poor association of heart defects with the ‘jogging ortholog’ gene list. This may simply be due to the lack of detection of *situs inversus totalis* ^53^, or reflect a redundancy in gene function, or it may be that the majority of mutations in these genes are simply not detected in humans because they are masked by first trimester spontaneous abortions, which are known to have a high level of heart defects ^48^.

The impact of lethal mutations on detection of genes associated with heart development would suggest that mutations in these genes would only be detected in individuals with mutations with relatively minor impact on gene function. This idea is supported by the recent identification of multiple ‘minor’ heterozygous mutations within a functional network in three patients with transposition of the great arteries. All of these genes either participate or cooperate within the Nodal signaling pathway ^54^ and the carriers of single mutations exhibit no heart or laterality defects. The impact of ‘minor’ mutations, such as these, may explain the contribution of ‘genetic modifiers’ to congenital heart defects with variable penetrance within a family ^55^, or may suggest a polygenic basis for some of these diseases ^56^. This is supported by model organism data, which provides evidence of multigenic origins for congenital heart disease ^56^. However, model organisms are rarely used to examine the impact of genetic modifiers on heart development, as the majority of model organisms are inbred and examination of mutations leading to ‘minor’ phenotypic variations is often not viewed with the same level of interest as the more extreme heart development defects.

Next Generation Sequencing (NGS) has the potential to identity many more instances of multiple mutations in genes which are functionally linked through a specific pathway. However, teasing out which gene mutations are contributing to a disease, as a genetic modifier or as the causative gene variant, and which are not involved in the disease, is likely to take considerable time. Gene Ontology, KEGG and Reactome pathways, along with protein interaction networks have the potential to inform the process of identifying genetic variants associated with heart defect risk through the identification of pathways and networks which are common to the genes associated with the risk gene variants. Consequently, interpretation of NGS data will be greatly improved with full annotation of the candidate genes involved. The identification of these risk gene variants is likely to be of considerable value to those patients seeking prenatal diagnosis. In addition, the identification of more genes associated with heart defects will also help clarify the conserved and divergent heart development pathways that exist between humans and key model organisms.

## Conclusions

This study demonstrates that full annotation, using GO, of a set of genes known to be associated with early stages of heart development in zebrafish can be used to confirm functional conservation of the role of these genes in a variety of developmental processes. While this study supports the assertion of gene function based on orthology between genes, it also identifies that for some genes there is no direct evidence for their conserved involvement in specific developmental processes through evolution. Consequently, for evolutionary studies, manual annotation of the genome of individual species will be necessary to enable a bioinformatics approach to investigating the evolution of developmental processes.
