# Supplementary material for: From zebrafish heart jogging genes to mouse and human orthologs: using Gene Ontology to investigate mammalian heart development
Source: F1000Res. 2014 Feb 10;2:242. Originally published 2013 Nov 13. [Version 2] doi: 10.12688/f1000research.2-242.v2 (PMC3931453; doi:10.12688/f1000research.2-242.v2)
Supplement: Peer review history

# Peer review: Referee response for version 2

**Published:** 2014-02-12

**Authors:** Jeroen Bakkers

**Affiliations:** 1 Cardiac Development and Genetics, Hubrecht Institute, Utrecht, Netherlands

I have read this submission. I believe that I have an appropriate level of expertise to confirm that it is of an acceptable scientific standard.

# Peer review: Referee response for version 1

**Published:** 2014-01-29

**Authors:** Jeroen Bakkers

**Affiliations:** 1 Cardiac Development and Genetics, Hubrecht Institute, Utrecht, Netherlands

The title and abstract accurately describes the content of the manuscript.

 

**Method:**

The analysis described here is based on the selected 30 genes from literature that are associated with the GO term ‘heart jogging’. According to the description by the authors these genes are involved in zebrafish heart jogging, but a better definition would be that these genes affect heart jogging (either direct or indirect). Indeed some of the genes that were included play a role in cilia function and establishment of the left-right axis.

Many more genes have been identified in zebrafish that affect left-right patterning and thus ‘heart jogging’. It remains unclear why these were not included in the search. The observation that many zebrafish left-right genes are not associated with the GO term ‘cardiac jogging’ could be due to annotation issues (e.g. not every authors uses the term ‘jogging’ for heart laterality defects in zebrafish embryos). This limitation in the design of this study should be discussed more thoroughly.

In addition the authors could make some conclusions about the usefulness of the GO term ‘heart jogging’ . Although heart jogging is a process specific to zebrafish it is controlled by a conserved left-right patterning mechanism. However the GO term ‘cardiac jogging’ is associated with genes that control heart morphogenesis and left-right patterning.

I have read this submission. I believe that I have an appropriate level of expertise to confirm that it is of an acceptable scientific standard.

# Response

**Published:** 2014-02-19

**Authors:** Ruth Lovering

**Affiliations:** University College London, UK

Dear Jeroen,

Thank you for your comments about our paper.

We have updated the methods, as suggested, to: A list of 30 zebrafish genes that affect heart jogging…

As already described in the methods we only included genes in this list where there was experimental evidence in ZFIN database which confirmed that these genes had an impact on heart jogging in zebrafish. Many genes are likely to be missing from this list because the heart jogging process is often not studied in zebrafish carrying mutations in relevant genes.

We have added the following statement to the methods:

This list does not represent all genes which play a role in heart jogging, as the process of heart jogging is not always studied in zebrafish carrying mutations in relevant genes.

We have not provided a detailed discussion about the GO term ‘heart jogging’ as we do not want to describe the detail of GO transitivity any further here.

Regards

Ruth

# Peer review: Referee response for version 1

**Published:** 2013-11-29

**Authors:** Vincent VanBuren

**Affiliations:** 1 Computational Systems Biology, Texas A&M University, Temple, TX, USA

The title is fine, but it would be better if it summarized findings rather than summarized the procedures. The abstract is an adequate summary of the article.

Required revisions:

- Methods- Generation of the list of zebrafish jogging genes: " *The search identified a further 23 zebrafish genes, however manual review of these publications led to 5 being disregarded, as the evidence for an involvement in heart jogging was not strong enough*." The phrase, " *was not strong enough*" does not provide sufficient detail to make construction of this list reproducible. The details should be provided.
   
- Discussion- paragraph 4: " *only a few of the 'jogging ortholog' genes, CCDC103, CCDC40, DNAAF1, LRRC6, NPHP3, and PKD2, are associated with heart looping defects, and thus provide evidence which suggests an involvement of these genesis left-right asymmetry determination in the heart*." First, making an assertion that genes are not associated with a particular process requires that high-powered studies were performed to reach a negative conclusion. Does the literature support this? Second, while it is true that eliminating one possibility increases the probability of other explanations, it is not very solid evidence for a particular explanation. The logic of the above statement should be better supported, or the statement should be removed.
   
- Discussion- paragraph 4: " *However, there are numerous other reasons why there is a poor association of heart defects with the 'jogging ortholog' gene list.*" This should be, "… *numerous other possible reasons*…".

I have read this submission. I believe that I have an appropriate level of expertise to confirm that it is of an acceptable scientific standard.

# Response

**Published:** 2014-02-19

**Authors:** Ruth Lovering

**Affiliations:** University College London, UK

Dear Vincent,

 

Thank you for your helpful comments. We also recognise that since the original search was conducted that there are now an additional 7 other zebrafish genes (Celf1, Cdc14aa, Cdc14b, Dmrt2a, Enpp2, Grem2, Ipar3) with experimental evidence supporting their role in heart jogging ( Matsui, T. *et al.,* 2012; Clément, A. *et al.*, 2012; Lai, S.L. *et al*., 2012; Müller, I.I. *et al.*, 2013)

 

As this comment will remain associated with this manuscript we will not add a comment about these additional genes to the manuscript itself, but we will revise the manuscript as follows:

 

**Methods - Generation of the list of zebrafish jogging genes section:**

To make it clearer how this list of zebrafish jogging genes was generated we will replace part of this section with the following: 

" *A list of 30 zebrafish heart jogging genes was compiled using a variety of approaches. Twelve zebrafish proteins were identified as they were already annotated to the ‘heart jogging’ GO terms, the remaining 18 proteins were then identified using the ZFIN ( http://zfin.org/) Site Search, with the search phrase 'heart jogging', and filtering using the 'Expression/Phenotypes' category. This search retrieves figures from papers that have ‘heart jogging’ in the figure legend, and thus are likely to be describing specific zebrafish genes (and proteins) involved in this process.  Many of these genes had not not yet been curated with GO terms. Each of the papers identified in this way were reviewed; of the 23 zebrafish genes identified in these papers five (Bmpr1aa, Tbx1, unm_hu119, unm_hu202, unm_hu304) were eliminated, as none of these papers provided experimental evidence for the involvement of these genes in heart jogging."*

 

**In Discussion - paragraph 4:**

We did not intend to make a negative conclusion here, this statement was making a positive statement that some of the 'jogging ortholog' genes, CCDC103, CCDC40, DNAAF1, LRRC6, NPHP3, and PKD2, are associated with heart looping defects. And as included in the comment below we do state that ‘there are numerous other reasons why there is a poor association of heart defects with the 'jogging ortholog' gene list’. To make this statement less controversial, we will modify it as follows:

*"Mutations in several human genes have now been identified as causative of abnormal heart looping, such as ACVR2B, LEFTY2, GJA1 and ZIC3(49–52), and some of the ‘jogging ortholog’ genes (CCDC103, CCDC40, DNAAF1, LRRC6, NPHP3 and PKD2) are also associated with heart looping defects. Thus providing evidence to support an involvement of these genes in left-right asymmetry determination in the heart."*

 

**Discussion- paragraph 4 (2** **^nd^** **comment):** 

As suggested we will revise this to " *However, there are numerous other possible reasons why there is a poor association of heart defects with the 'jogging ortholog' gene list.*"
